# Draft genome sequence of *Thermococcus waiotapuensis* WT1^T^, a thermophilic sulfur-dependent archaeon from the order *Thermococcales*

**DOI:** 10.1128/mra.00815-23

**Published:** 2023-12-14

**Authors:** Sarah H. Manners, Carlo R. Carere, Manpreet K. Dhami, Renwick C. J. Dobson, Matthew B. Stott

**Affiliations:** 1 Te Kura Pūtaiao Koiora School of Biological Sciences, Te Whare Wānanga o Waitaha University of Canterbury, Christchurch, New Zealand; 2 Biomolecular Interaction Centre, Te Whare Wānanga o Waitaha, University of Canterbury, Christchurch, New Zealand; 3 Department of Chemical and Process Engineering, Te Tari Pūhanga Tukanga Matū, Te Whare Wānanga o Waitaha, University of Canterbury, Christchurch, New Zealand; 4 Biocontrol and Molecular Ecology, Manaaki Whenua Landcare Research, Lincoln, New Zealand; Portland State University, Portland, Oregon, USA

**Keywords:** *Thermococcales*, *Euryarchaeota*, Aotearoa-New Zealand, thermophile, sulfur-utilizing, archaea

## Abstract

*Thermococcus waiotapuensis* WT1^T^ is a thermophilic, peptide, and amino acid-fermenting archaeon from the order *Thermococcales*. It was isolated from Waiotapu, Aotearoa-New Zealand, and has a genome size of 1.80 Mbp. The genome contains 2,000 total genes, of which 1,913 encode proteins and 46 encode tRNA.

## ANNOUNCEMENT

The majority of described *Thermococcus* spp. have been isolated from marine hydrothermal vents (1 – 5) and are characterized by their thermophilic, peptide, and amino acid-fermenting phenotype. By contrast, only two described species, *Thermococcus waiotapuensis* WT1^T^ (6) and *Thermococcus zilligii* AN1 (7) have been found in freshwater hot spring environments. *Thermococcus waiotapuensis* WT1^T^ was isolated from Champagne Pool at Waiotapu (Aotearoa-New Zealand) by González et al. (6) and is one of the only known representatives of freshwater inhabitants within the genus and is thus of interest for whole-genome sequencing. The *iwi* (Māori tribe) Ngāti Tahu-Ngāti Whaoa are *kaitiaki* (guardians) of Waiotapu and we acknowledge their *mana whenua* (customary rights) over the archaeon, its genome and the location in which it was isolated. All members of the *Thermococcales* order are obligate heterotrophs, strictly anaerobic, grow at temperatures between 50 and 100°C, and are dependent on elemental sulfur for growth (8).

Here we report the draft genome sequence of *T. waiotapuensis* WT1^T^. WT1^T^ was obtained from the Japan Collection of Microorganisms (JCM 10985) and cultivated for 3 weeks anaerobically at 70°C in the medium as described by González et al. (6). Genomic DNA was extracted using the Nucleospin Microbial DNA kit (Macherey Nagel, Duren, Germany) according to the manufacturer’s instructions. The sequencing library was prepared for 150 bp paired-end reads with a final average library size of 478 bp. Sequencing adapters were added *via* ligation. The sequencing library was prepared according to the QIAGEN FX DNA Library Preparation Kit (QIAGEN, Hilden, Germany), and the quality control of the finished library was assessed *via* Agilent TapeStation (Agilent, Santa Clara, CA). Whole-genome shotgun sequencing was undertaken on the Illumina NovaSeq6000 platform (Illumina, San Diego, CA, USA). Default parameters were used for all assembly, quality control, and annotation software unless otherwise specified. Adapter trimming was performed using Trimmomatic (v0.36) (9) and read quality control with FastQC (v.0.1.12) (10). A total of 1.5-Gbp raw sequences (10.2 M paired-end reads) were assembled using SPAdes (v3.15.3) (11). The assembly was evaluated using QUAST (v4.4.0), and genome completeness was estimated using CheckM (v1.0.18) (12, 13). The genome was annotated *via* the NCBI Prokaryotic Genome Annotation Pipeline (PGAP; v6.6) (14) (Genbank accession no. JAVDZE000000000) using the GeneMarkS-2+ protein set. A functionally equivalent annotation of 11 scaffolds using the Integrated Microbial Genomes annotation pipeline v5.1.9 (15) is also available (IMG genome ID 8019721040).

The *T. waiotapuensis* WT1^T^ draft genome is 1,804,563 bp and is predicted to contain 2,000 total genes, of which 1,913 are predicted to encode proteins. The genome completeness was estimated at 100%, consisting of 11 contigs and the mol% G + C content is 53.42%. The N_50_ value was 442,258, the L_50_ was 2, and genome coverage was ~855× . The genome is predicted to encode 52 RNA genes, including 46 tRNAs, and a single copy of the 16S and the 23S rRNA, and two copies of the 5S rRNA. A pairwise, average nucleotide identity (ANI) analysis between *T. waiotapuensis* WT1^T^ and *T. zilligii* AN1 (GCF_000258515.1) using the pairwise ANI tool in the Integrated Microbial Genomes database (15) resulted in a percentage similarity score of ~96.23%. The genome is predicted to encode genes for amino acid transferases and glutamate dehydrogenase, which are both necessary for the fermentation of amino acids (16). Predicted Ni-Fe hydrogenase complexes and sulfhydrogenases are present in the genome, consistent with the general metabolic capability of *Thermococcus* spp (17 – 19).

## Data Availability

The assembled genome was deposited in the Genomes Online Database (20) (GOLD analysis ID Ga0604744), for associated annotation with the Integrated Microbial Genomes annotation pipeline (v5.1.9) (15) (IMG genome ID 8019721040). This Whole Genome Shotgun project has been deposited at DDBJ/ENA/GenBank under the accession JAVDZE000000000. The version described in this paper is version JAVDZE010000000. The sequencing reads are available under BioProject accession number PRJNA1006204 and the SRA under accession SRR25820473.
